# Comparison of bone single-photon emission computed tomography (SPECT)/CT and bone scintigraphy in assessing knee joints

**DOI:** 10.1186/s12880-021-00590-8

**Published:** 2021-03-26

**Authors:** Young-Sil An, Do Young Park, Byoung-Hyun Min, Su Jin Lee, Joon-Kee Yoon

**Affiliations:** 1 Department of Nuclear Medicine and Molecular Imaging, School of Medicine, Ajou University, 206, World cup-ro, Yeongtong-gu, Suwon-si, Suwon, Gyeonggi-do 16499 Korea; 2 Department of Orthopedic Surgery, Ajou University School of Medicine, Suwon, Korea

**Keywords:** Single photon emission computed tomography/computed tomography, SPECT/CT, Bone scintigraphy, Knee joint, Standardized uptake value, Quantitative evaluation

## Abstract

**Background:**

This study attempted to compare the radiopharmaceutical uptake findings of planar bone scintigraphy (BS) and single photon emission computed tomography (SPECT)/computed tomography (CT) performed on knee joints.

**Methods:**

We retrospectively included 104 patients who underwent bone SPECT/CT and BS 4 h after the intravenous administration of technetium-99m-hydroxymethylene diphosphonate (^99m^Tc-HDP) for pain in the knee joint. The uptake degree of each of the knee regions (medial femoral, lateral femoral, medial tibial, lateral tibial, and patellar area) in planar images and SPECT/CT were evaluated by visual (grades 0 to 2) and quantitative analyses (uptake counts for planar image and standardized uptake values [SUVs] for SPECT/CT).

**Results:**

The uptake grades assessed visually on the planar images differed significantly from the uptake grades on SPECT/CT images in all areas of the knee (all *p* < 0.001), and SPECT/CT imaging revealed a larger number of uptake lesions than those noted in planar imaging for each patient (3.3 ± 2.0 vs 2.4 ± 2.3, *p* < 0.0001). In all regions of the knee, all of the quantitative values, including uptake counts obtained from the planar image as well as the maximum SUV (SUVmax) and mean SUV (SUVmean) obtained from SPECT/CT, showed statistically higher values as their visual grades increased (all *p* < 0.001). However, when analyzed for each area, only the SUVmax showed a significant difference by grade in all knee regions. Quantitative uptake values obtained from planar images were moderately correlated with SUVs of SPECT/CT images (*r* = 0.58 for SUVmean and *r* = 0.53 for SUVmax, all *p* < 0.001) in the total knee regions. Looking at each area, there was a significant but low correlation between the uptake counts of the planar images and the SUVs on SPECT/CT in the right lateral tibial region (*r* = 0.45 for SUVmean, *r* = 0.31 for SUVmax, all *p* < 0.001).

**Conclusions:**

In assessing knee joints, the findings of planar images and SPECT/CT images differ both visually and quantitatively, and more lesions can be found in SPECT/CT than in the planar images. The SUVmax could be a reliable value to evaluate knee joint uptake activity.

## Background

Bone scintigraphy (BS) using a Tc-99m labeled bone-seeking radiopharmaceutical, which actively deposited in the areas with new bone formation and/or increased blood flow [1], has often been used in clinical practice to evaluate the knee joint problems of patients. However, BS can only obtain planar images, which have shown limitations in evaluating the deep part of the knee joint or determining the specific anatomical location of the abnormal uptake site of the radiopharmaceutical [2]. This study is an attempt to compensate for this limitation of BS using hybrid single photon emission computed tomography (SPECT)/computed tomography (CT) combined with functional SPECT imaging and anatomic CT imaging [3].

Previous studies reported that SPECT/CT could be a helpful tool for precise localization and accuracy of characterization in assessing knee joints [2, 4–8]. Also, there are a few previous studies that quantitatively analyzed the uptake of radiopharmaceuticals in knee SPECT/CT [5, 9], as it is possible to measure the uptake of radiopharmaceuticals as in positron emission tomography (PET) using standardized uptake values (SUVs) [10]. Although BS can be quantified using the traditional method in the form of counts per second, this is fundamentally different from SUVs [11].

At a time when the clinical use of SPECT/CT for knee joints is expected to increase, we have raised questions about how SPECT/CT is different from BS and whether SUVs obtained from SPECT/CT really have the reliability to be used in reading images. However, previous studies comparing BS and SPECT/CT directly in knee joints are very limited [2]. In particular, there are few studies comparing the quantified uptake value in planar BS with SUVs in SPECT/CT in knee joints.

The purpose of this study is to directly compare BS and SPECT/CT findings performed at the knee joint to determine their qualitative and quantitative differences.

## Materials and methods

### Subjects

From June 2019 to December 2019, 104 patients (19 men, 85 women, mean ± standard deviation [SD] age 58.3 ± 9.0 years) who underwent whole body bone scintigraphy (WBBS) and bone SPECT/CT to evaluate knee pain at our single institution were included in this study. Patients with metallic materials in the knee that could cause problems in CT-based attenuation correction when analyzing SPECT images and patients with a history of previous knee surgery were excluded from this study. This retrospective study was conducted in accordance to the guidelines of the Declaration of Helsinki and approved by the Institutional Review Board of Ajou University (MED-MDB-19–475), through which informed consent was waived.

### BS and bone SPECT/CT acquisition

WBBS was performed 4 h after the injection of 740 MBq technetium-99m hydroxymethylene diphosphonate (^99m^Tc-HDP). Anterior and posterior views were acquired with a speed of 20 cm/min, zoom of 1.0, pixel size of 2.2 mm, and resolution of 256 × 1024 matrix, using a double-headed gamma camera equipped with a NMCT/670 low-energy high-resolution collimator (GE Healthcare, Pittsburgh, PA, USA). Subsequently, SPECT/CT images of both knees were obtained using the NMCT/670 SPECT/CT scanner (GE Healthcare). SPECT images over the knee area were acquired using step-and-shoot mode (10 s/step, 4 angular increment, total 90 steps) and zoom factor of 1.14 with a photon peak of 140.5 keV (10% window) and a scatter window of 120 keV (5% window). A CT image without any contrast was acquired using a tube voltage of 120 kVp with a tube current of auto mA (60–210 mA). An adaptive statistical iterative reconstruction algorithm (ASiR, GE Healthcare) was used to reconstruct CT images into 2.5-mm-thick transaxial slices with 512 × 512 image matrix. SPECT images were processed using automated motion correction, CT-based attenuation correction, scatter correction, and resolution recovery using the Volumetrix MI Evolution for Bone (GE Healthcare) software program [12]. Reconstruction of the SPECT image was performed in different ways depending on purpose. For visual analysis, ordered-subset expectation maximization (OSEM) with 2 iterations, and 10 subsets was used for image reconstruction with Butterworth post-filtering (frequency of 0.60 cycles/cm, power 10), and the images were displayed as a 128 × 128 matrix with a section thickness of 3.88 mm. For quantitative analysis, the images were reconstructed using OSEM with 4 iterations and 10 subsets without post-filtering, which is a proven optimized reconstruction method for quantification [13–15].

### Visual analysis of images

**Fig. 1:**
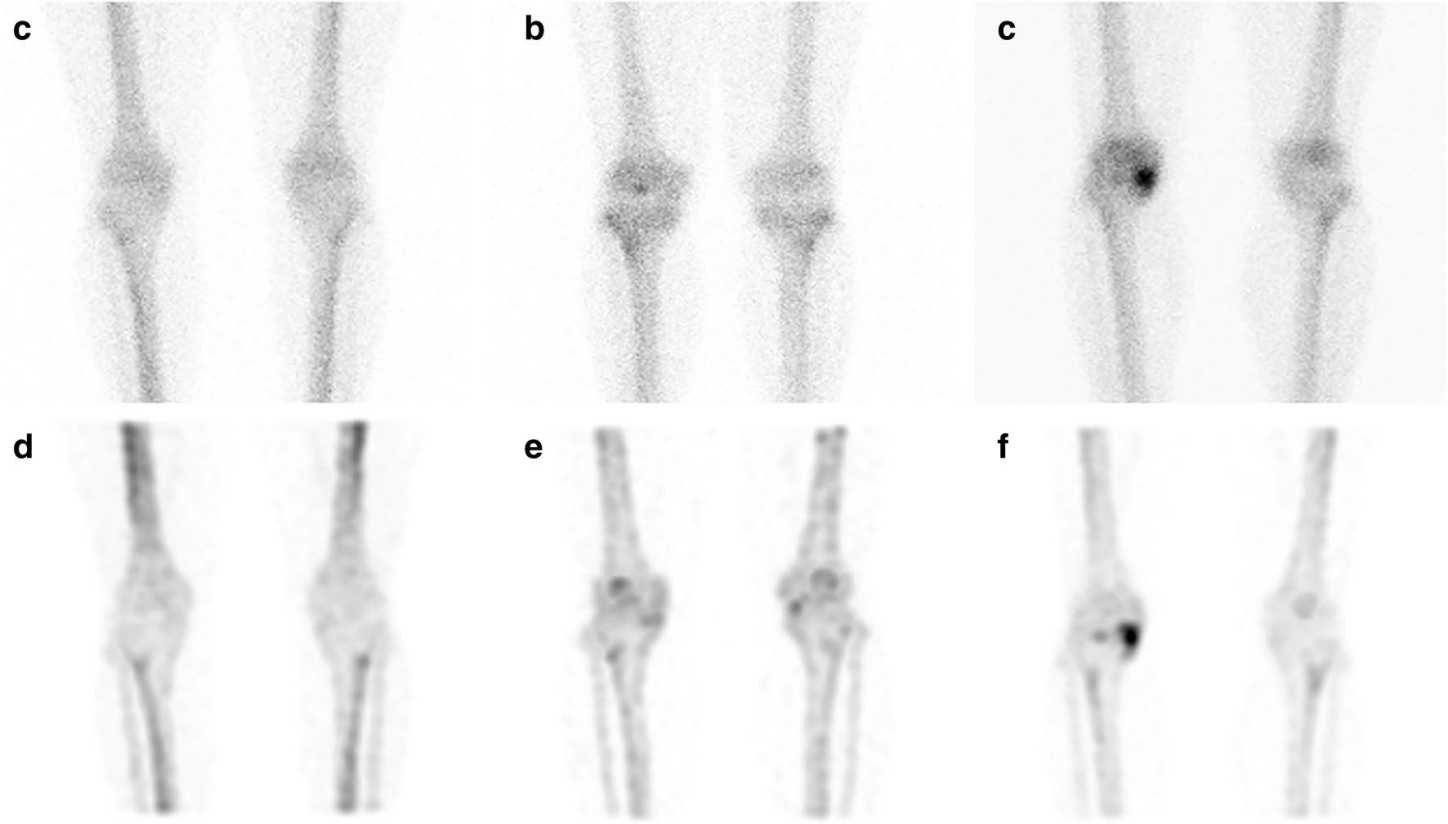
Representative image of the visual grading of radiopharmaceutical uptake. In the planar images, cases where no significant uptake was observed in the knee were classified as grade 0 (**a**), grade 1 when uptake was mild (**b**), and grade 2 when uptake was intense (**c**). In the SPECT images, similarly, grade 0 signified that no meaningful uptake was observed visually (**d**), grade 1 denoted mild uptake (**e**), and grade 2 was when intense uptake was observed (**f**)

An expert (YA) with 15 years of experience in nuclear medicine visually evaluated the knee area in the WBBS and knee bone SPECT/CT images. In each image, both knees were divided into medial femoral, lateral femoral, medial tibial, lateral tibial, and patellar regions, and the expert graded the uptake of radiopharmaceuticals from 0 to 2 (0 = no uptake, 1 = mild, 2 = intense). The uptake was considered significant uptake when the uptake grade was 1 or higher, and the total number of sites showing significant uptake per patient was recorded for each planar and SPECT image. The degree of uptake in the patellofemoral joint area was evaluated only in the SPECT/CT image and this region was not included in the total number of sites showing significant uptake. Figure 1 shows a representative case in which the degree of radiopharmaceutical uptake was visually graded in planar and SPECT/CT images.

### Quantitative analysis of images

**Fig. 2:**
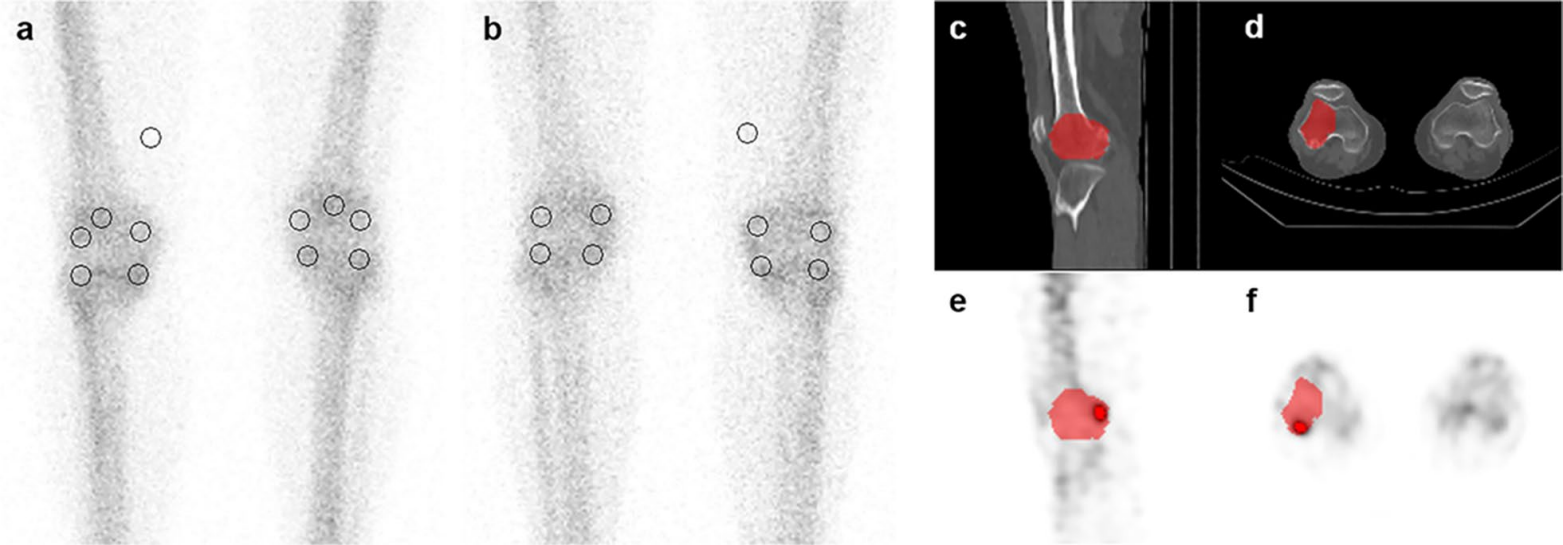
Representative images of regions of interest (ROIs) placements for quantitative analysis. In the anterior (**a**) and posterior (**b**) view of the knee plane image, circular ROIs 15 mm in diameter were drawn on the medial femoral, lateral femoral, medial tibial, lateral tibial, and patella regions, and the same size of ROI was set on the right thigh soft tissue as a reference region. In the SPECT/CT images, volumes of interest (VOIs, red marked regions of **c** and **d** images) were manually drawn in the regions corresponding to the right lateral femoral region in CT (c: sagittal image, d: transaxial image). This drawn VOI was projected on the SPECT image (**e** sagittal image, **f** transaxial image) of the same area, and the VOI (red marked part of e and f image) was set

Images were analyzed on a Xeleris Workstation (GE Healthcare, Milwaukee, WI, USA). For quantitative analysis in planar images, circular regions of interest (ROIs) were set in the medial femoral, lateral femoral, medial tibial, and lateral tibial regions in the anterior and posterior views for each side. Patellar ROI was drawn only in the anterior view, and for background correction, circular ROIs of the same size (15 mm in diameter) were set in the soft tissue area of the right thigh in the anterior and posterior views as reference areas (Fig. 2a, b). The background corrected value was obtained by dividing the mean count of each region by the mean count of the reference, and the geometric mean values calculated from the anterior and posterior counts [16] were used as the final analysis data. There was one exception to this procedure: the uptake of the patella was determined by the mean count obtained from only the anterior view.

For quantification of SPECT/CT images, SPECT data were processed with iterative reconstruction, attenuation correction, scatter correction, and resolution recovery and prepared through the Preparation for Q.Metrix program (GE Healthcare) used for the Q.Metrix software (GE Healthcare). When the volumes of interest (VOIs) in the areas corresponding to the medial femoral, lateral femoral, medial tibial, lateral tibial, and patellar regions of both knees were manually drawn on CT images, the same area was projected on the SPECT image (Fig. 2c–f). Corresponding maximum and mean SUVs (SUVmax and SUVmean) in this area, corrected by body weight, were recorded.

### Statistical analysis

All statistical analyses were performed using MedCalc software (version 19.2.1; MedCalc Software bvba, Ostend, Belgium). A power analysis was used to calculate the sample size required for this study using a significance (α) level of 5% and statistical power (1 − β) of 80%. A sample size of 78 was required to obtain an appropriate confidence level; thus, the sample size finally achieved (*n* = 104) was sufficient.

All of the continuous variables included in this study had normal distributions verified by the Kolmogorov–Smirnov test, and the values are expressed as the means and SDs. Using the chi-square test, we examined whether the visually evaluated uptake grades of radiopharmaceuticals on the planar and the SPECT/CT images differed. Paired sample t-tests were used to analyze whether there was a difference between the total number of significant uptakes found in the planar images and the number found in the SPECT/CT images. Whether the quantitative values differed according to the visually evaluated uptake grade of radiopharmaceuticals was analyzed using one-way analysis of variance (ANOVA) followed by the Tukey–Kramer post hoc test for pairwise comparisons of multiple groups. The Pearson's correlation coefficient test was used to analyze the correlation between quantitation values in planar images and SUVs from SPECT/CT images. The magnitude of the correlation was interpreted as negligible ($\left| r \right|$ < 0.3), low ($\left| r \right|$ = 0.30–0.49), moderate ($\left| r \right|$ = 0.50–0.69), high ($\left| r \right|$ = 0.70–0.89), or very high ($\left| r \right|$ ≥ 0.90) [17]. *p* values < 0.05 were considered significant.

## Results

### Results of visual analysis

**Fig. 3:**
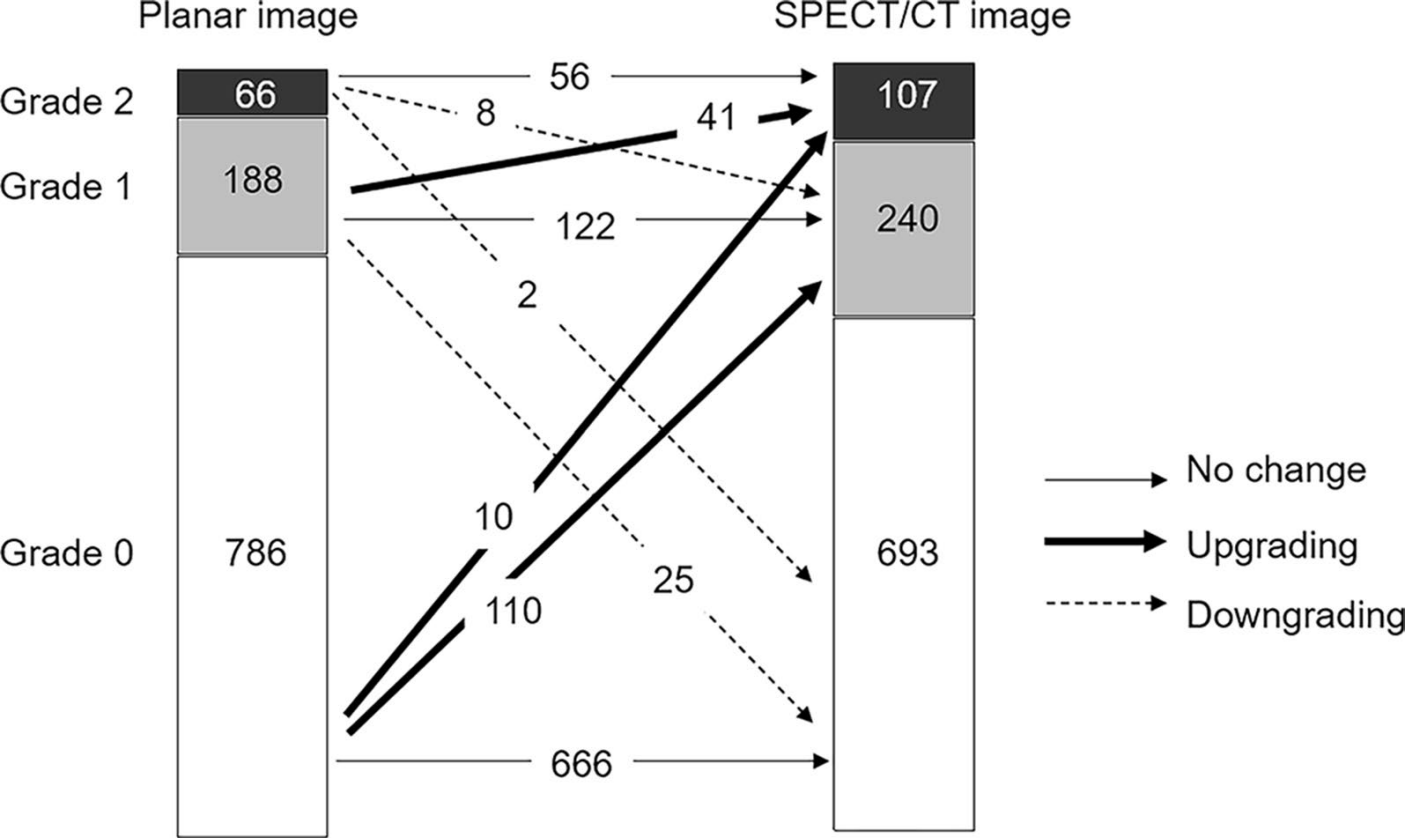
Distribution of grades according to the planar and SPECT/CT images. The uptake levels in 161 areas (15.5%) evaluated by planar images were upgraded in the SPECT/CT images, and those in 35 regions (3.4%) were downgraded in the SPECT/CT images

The visual uptake grades in the planar images showed a significant difference from those of the SPECT/CT images and in all parts of the knee (all *p* < 0.0001, Table 1). About 80% (844/1040) of grades in the planar images were the same as those in the SPECT/CT images, but 15.5% (161/1040) showed upgrade results by SPECT/CT image. Detailed grade distributions and grade changes are shown in Fig. 3. The total number of sites per patient with visually significant uptake (grade 1 or higher) showed a statistically significant difference, with 2.4 ± 2.3 in the planar images and 3.3 ± 2.0 in the SPECT/CT images (*p* < 0.0001). In the patellofemoral joints, 23 lesions (11%, 23/208) with uptakes of grade 1 or higher were found according to the SPECT/CT images.

**Table 1:** Comparison of uptake grades evaluated visually in planar and SPECT/CT images

Region of knee	Grade in planar image (n, %) Grade 0/Grade 1/Grade 2	Grade in SPECT/CT image (n, %) Grade 0/Grade 1/Grade 2	*p* value
Left lateral femur	80 (76.9%)/20 (19.2%)/4 (3.8%)	77 (74.0%)/20 (19.2%)/7 (6.7%)	< 0.0001
Left lateral tibia	84 (80.8%)/18 (17.3%)/2 (1.9%)	84 (80.8%)/14 (13.5%)/6 (5.8%)	< 0.0001
Left medial femur	70 (67.3%)/27 (26.0%)/7 (6.7%)	57 (54.8%)/32 (30.8%)/15 (14.4%)	< 0.0001
Left medial tibia	72 (69.2%)/17 (16.3%)/15 (14.4%)	64 (61.5%)/20 (19.2%)/20 (19.2%)	< 0.0001
Left patella	76 (73.1%)/26 (25.0%)/2 (1.9%)	59 (56.7%)/40 (38.5%)/5 (4.8%)	< 0.0001
Right lateral femur	84 (80.8%)/15 (14.4%)/5 (4.8%)	75 (72.1%)/20 (19.2%)/9 (8.7%)	< 0.0001
Right lateral tibia	96 (92.3%)/8 (7.7%)/0 (0%)	88 (84.6%)/14 (13.5%)/2 (1.9%)	< 0.0001
Right medial femur	75 (72.1%)/18 (17.3%)/11 (10.6%)	63 (60.6%)/22 (21.2%)/19 (18.3%)	< 0.0001
Right medial tibia	74 (71.2%)/14 (13.5%)/16 (15.4%)	65 (62.5%)/24 (23.1%)/15 (14.4%)	< 0.0001
Right patella	75 (72.1%)/25 (24.0%)/4 (3.8%)	61 (58.7%)/34 (32.7%)/9 (8.7%)	< 0.0001
Total	786 (75.6%)/188 (18.1%)/66 (6.3%)	693 (66.6%)/240 (23.1%)/107 (10.3%)	< 0.0001

### Results of quantitative analysis

**Fig. 4:**
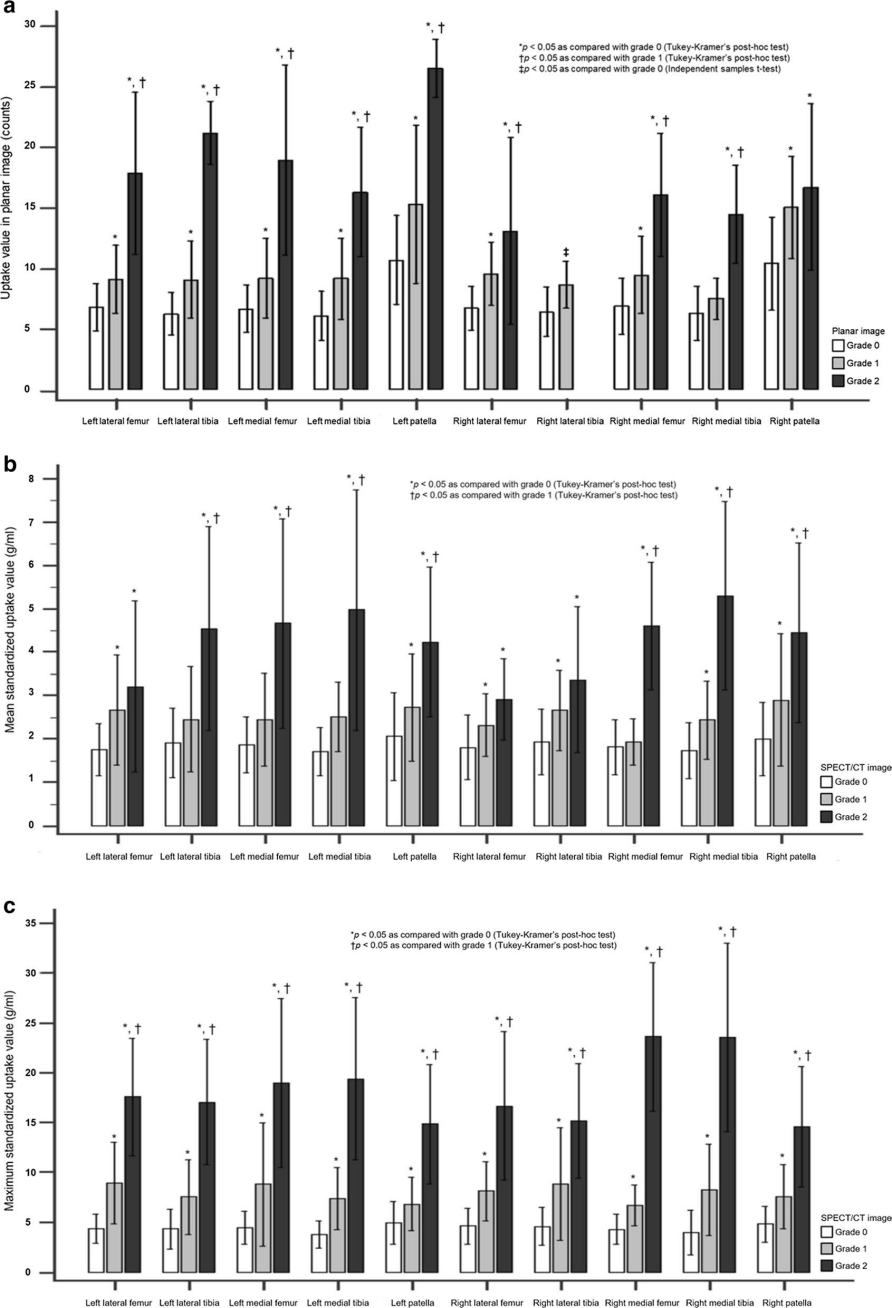
Comparison of quantitative values according to the visually evaluated uptake grades. The uptake values in planar images showed significant differences according to visual grade (**a**). Significant differences in values were observed between all grades except in the right medial tibial and right patella regions, according to post-hoc tests. SUVmean differed significantly according to the grades visually assessed in SPECT/CT (**b**), and a significant difference between all grades was shown in the post-hoc test only in the areas of both patellae and the right medial tibia. On the other hand, SUVmax had significant differences in all grades in all areas, according to post-hoc tests (**c**)

In total, for regions on the planar images, the quantitative uptake values of 786 areas corresponding to visual grade 0 were 7.33 ± 2.91, the uptake values of grade 1 (*n* = 188) was 10.73 ± 4.65, and those of grade 2 (*n* = 66) were 16.46 ± 5.83, showing a significant uptake difference between grades (*p* < 0.001 in ANOVA test, *p* < 0.05 in post-hoc test between all grades). When looking at each area separately, the uptake values also showed a significant difference between grades (all *p* < 0.001 in ANOVA tests except for the right lateral tibia [*p* = 0.003] using an independent sample t-test due to the absence of grade 2, Fig. 4a). As a result of the post-hoc test, in most areas except right medial tibia and right patella, as the visual grade increased, the uptake value also increased significantly. The uptake value failed to show a significant difference between grade 0 and grade 1 in the right medial tibial region or between grade 1 and grade 2 in the right patella (Fig. 4a).

In all regions of the SPECT/CT image, the SUVmean of visual grade 0 (*n* = 693) was the lowest with 1.86 ± 0.74; grade 1 (*n* = 240) was 2.54 ± 1.11, grade 2 (*n* = 107) was 4.50 ± 2.16, and the SUVmean showed statistically significant differences between visual grades (*p* < 0.001 in ANOVA test, *p* < 0.05 in post-hoc test between all grades). The SUVmean also showed a significant difference according to visual grade in each area of the knees (*p* < 0.001 in all ANOVA tests, Fig. 4b). The SUVmean values of both patellae and the right medial tibial region showed significant differences between all grades, but there was no significant difference in SUVmean between grade 0 and grade 1 in either the medial femur or left tibia, or between grade 1 and grade 2 in either the lateral femur or the right lateral tibial region (Fig. 4b).

In SUVmax values of all knee regions, grade 2 (*n* = 107) showed the highest value (19.53 ± 8.06), followed by grade 1 (*n* = 240, 7.84 ± 3.98) and grade 0 (*n* = 693, 4.45 ± 1.83), and these were statistically significant differences (*p* < 0.001 in ANOVA test, *p* < 0.05 in post-hoc test between all grades). Even in each area of knee, there were also significant differences between them (*p* < 0.001 in all ANOVA tests, Fig. 4c), and the post-hoc test results also showed significant differences between all grades in all areas (Fig. 4c).

### Correlation of quantitative values in planar and SPECT/CT images

**Fig. 5:**
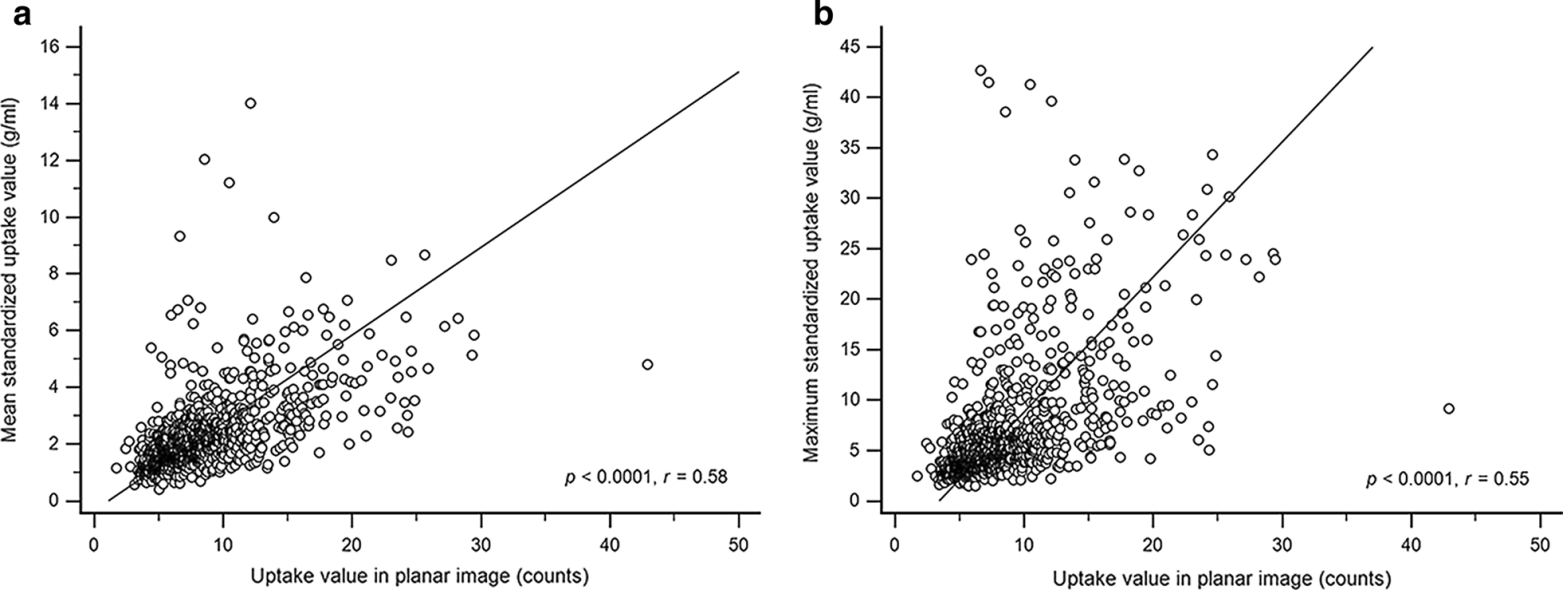
Scatter diagram of correlation between uptake values in planar images and SUVs from SPECT/CT images. Quantitative uptake values in planar images showed significant correlation with SUVs [SUVmean (**a**), SUVmax (**b**)], and their correlation was moderate

Quantitative uptake values obtained from planar images and SPECT/CT images showed a significant correlation in all knee regions (all *p* < 0.0001, Table 2). There was a moderate correlation between planar uptake values and SUVs for the total knee area (*r* = 0.58 between SUVmean and planar uptake values, *r* = 0.53 between SUVmax and planar uptake, Fig. 5).

**Table 2:** Correlation of quantitative values between planar and SPECT/CT images

Region of knee	Uptake value in planar image	SUVmean	Correlation coefficient *r* (95% CI) between uptake value in planar image and SUVmean	Correlation *p* value between uptake value in planar image and SUVmean	SUVmax	Correlation coefficient *r* (95% CI) between uptake value in planar image and SUVmax	Correlation *p* value between uptake value in planar image and SUVmax
Left lateral femur	7.68 ± 3.28	2.03 ± 1.01	0.66 (0.53–0.76)	< 0.0001	6.17 ± 4.42	0.61 (0.47–0.71)	< 0.0001
Left lateral tibia	7.07 ± 3.06	2.14 ± 1.16	0.69 (0.57–0.78)	< 0.0001	5.54 ± 4.04	0.71 (0.60–0.79)	< 0.0001
Left medial femur	8.18 ± 4.29	2.45 ± 1.51	0.59 (0.45–0.70)	< 0.0001	7.90 ± 6.89	0.67 (0.54–0.76)	< 0.0001
Left medial tibia	8.10 ± 4.61	2.49 ± 1.82	0.63 (0.49–0.73)	< 0.0001	7.50 ± 7.15	0.78 (0.69–0.85)	< 0.0001
Left patella	12.17 ± 5.32	2.42 ± 1.24	0.57 (0.42–0.69)	< 0.0001	6.19 ± 3.37	0.53 (0.38–0.65)	< 0.0001
Right lateral femur	7.45 ± 2.90	2.01 ± 0.83	0.63 (0.50–0.74)	< 0.0001	6.36 ± 4.51	0.68 (0.56–0.77)	< 0.0001
Right lateral tibia	6.63 ± 2.07	2.05 ± 0.84	0.45 (0.38–0.59)	< 0.0001	5.39 ± 3.38	0.31 (0.13–0.48)	< 0.0001
Right medial femur	8.34 ± 4.01	2.35 ± 1.35	0.60 (0.46–0.71)	< 0.0001	8.37 ± 8.10	0.69 (0.57–0.78)	< 0.0001
Right medial tibia	7.76 ± 3.84	2.41 ± 1.61	0.66 (0.54–0.75)	< 0.0001	7.81 ± 8.08	0.66 (0.54–0.76)	< 0.0001
Right patella	11.80 ± 4.56	2.51 ± 1.42	0.63 (0.50–0.73)	< 0.0001	6.60 ± 3.97	0.53 (0.38–0.66)	< 0.0001
Total	8.52 ± 4.28	2.29 ± 1.32	0.58 (0.54–0.62)	< 0.0001	6.78 ± 5.75	0.55 (0.50–0.59)	< 0.0001

*CI* confidence interval, *SUVmean* mean standardized uptake value, *SUVmax* maximum standardized uptake value

Looking at each area, high correlations were apparent between the planar uptake value and the SUVmax in SPECT/CT images in the left medial tibial (*r* = 0.78) and femoral region (*r* = 0.71), while the right lateral tibia region showed a low correlation between the uptake value in the planar image and the SUVs (*r* = 0.45 for SUVmean, *r* = 0.31 for SUVmax). All other areas showed moderate correlation (Table 2).

## Discussion

We could find clear differences in visual findings between BS and bone SPECT/CT of the knee joints in our study. The visual uptake from planar BS was upgraded from a fairly high proportion of 15.5% through SPECT/CT image analysis. Also, it was interesting to note that there were some cases in which visual uptake evaluated with BS was actually downgraded by SPECT/CT, even though it was a small portion (3.4%, 35/1040). When we looked at the lesions downgraded by SPECT/CT in more detail, most lesions were patellar uptakes in the planar BS. They were identified by SPECT/CT as hot uptake lesions in the patellofemoral joints or in the femur close to the patella, not in the true patella. Therefore, it seems that the downgraded cases were the result of overcoming planar imaging limitations caused by summation and the increase in the accuracy of anatomic localization due to SPECT/CT.

Our study showed that SPECT/CT was able to detect a statistically greater number of hot uptake lesions than was planar BS in visual assessment. It may be only natural that SPECT/CT could find more hot uptake lesions than planar BS. However, it is not so easy to analyze and clearly prove this fact through research. In fact, we were able to find only one previous study comparing BS and bone SPECT/CT in the knee joints [2], and they reported that SPECT/CT was able to find significantly more lesions than BS in patients with knee pain. In fact, clinicians are hesitant to order SPECT/CT because it is more expensive, has a longer examination time, and may involve a greater radiation exposure than BS. In this situation, our findings might help clinicians to be confident that SPECT/CT can find more lesions than BS and could be the basis for justifying their ordering SPECT/CT.

Patellofemoral joints are difficult to evaluate with planar images, but can be evaluated in SPECT/CT, which provides 3D images of knee joints. A previous study by Hirschmann et al. [6] reported that when SPECT/CT was performed on patients with knee pain after total knee arthroplasty, patellofemoral joint lesions could be effectively differentiated from other knee compartments. Our results also showed visual hot uptake lesions in 23 of the 208 patellofemoral joints in enrolled patients. Thus, the clinical usefulness of SPECT/CT in evaluating patellofemoral joints is expected in the future.

As our result of analyzing the differences in quantitative values according to the visual grade, the uptake values of planar BS and the SUVmean values of SPECT/CT did not show significant differences in some of the visual grades; only the SUVmax showed a meaningful difference between all visual grades (grades 0, 1 and 2). The usefulness of the SUVmax obtained from SPECT/CT in evaluating activity in osteoarthritic disease of the knees has been demonstrated through previous studies by Kim et al. [9], and SUVmax has the advantage of being 100% reproducible regardless of the reader [11]. On the other hand, SUVmean varies depending on how the reader sets the ROI, and thus may be less reliable than the SUVmax, so SUVmax is preferred as a quantitative value for evaluating joints [18]. Furthermore, our study showed that SUVmax reflects visual grade well, so our results will serve as another basis for nuclear medicine physicians to no longer doubt or hesitate about the clinical use of SUVmax on knee joints.

We also compared the quantitative values in the planar images with those in the SPECT/CT images in our current study. When we planned the study, we expected to have a strong correlation between them, but the results did not meet this expectation. Quantitative values in planar images and SUVs from SPECT/CT showed significant correlations, but the degree of correlation was moderate in most parts of the knee. There was even a knee region with low correlation (the right lateral tibial region). As far as we know, there are few previous studies that have reported the association of SUVs in SPECT/CT with quantitative values in planar images at knee joints. The SUVs with the radioactivity per volume units generated from SPECT/CT imaging were fundamentally different from the quantification values of counts per second units obtained from BS [11, 19]. For this reason, it is expected that their correlation was not very strong. Here, we would like to suggest that the quantitative values obtained from the planar images and the SUVs obtained from SPECT/CT may be correlated with each other, but the degree of correlation is not very strong.

In our study, when it was considered normal to visually show grade 0, the normal SUVmax was 4.45 ± 1.83 and the SUVmean was 1.86 ± 0.74 at the knee joints. Although these values seem to be similar to the median SUV of 3.2 of normal limb bone suggested in a previous study of Arvola et al. [20], it is difficult to compare these values because the study design is different from ours. However, our study contains a relatively large sample size (*n* = 104) compared to the previous study (*n* = 29), so we think that our study has strengths in this regard. If the clinicians want to use SUVs when reading bone SPECT/CT performed on knee joints, we expect that it will be clinically helpful to refer to the normal values of SUVs that we suggest.

The study has some limitations as follows. First, this study did not use the patients’ clinical information, including the patients’ symptoms, for analysis. We excluded the clinical symptoms of patients from the analysis because the main goal was to compare the image findings of planar BS images with SPECT/CT in this study. This may be a limitation of this study, but it was due to our effort to conduct an objective analysis by excluding external factors as much as possible. In addition, a previous study revealed that the patients’ symptoms were not associated with SUVmax in knee joints [9], so this limitation is unlikely to be a major problem. Another limitation is that our study did not include the findings of SPECT images separately. In the previous study by Lu et al. [2], BS, SPECT, and SPECT/CT were compared in knee joints, but this study lacked SPECT findings. We considered whether to include SPECT-only findings without CT imaging in our research at the beginning of the study, but our study was designed to focus on SUV, the quantification value that can be obtained from SPECT/CT, and SPECT-only findings were not included in our study because SPECT alone cannot provide SUVs. We believe that this limitation did not affect the main point of our study. As a final limitation, this study did not consider lateral planar view data of the knee. This retrospective study could not include lateral view data, because only anterior and posterior planar views were obtained to avoid patient discomfort due to prolonged examination time in our institution's routine protocol. Since the knee lateral planar view can be helpful in evaluating lesions of the patella and patellofemoral joints [21], future studies including this data are needed.

## Conclusions

Planar BS and bone SPECT/CT images performed at knee joints clearly differed when assessed visually and quantitatively, and SPECT/CT was able to detect more lesions than BS. The SUVmax could be a robust reliable value for quantifying and evaluating uptake activity of the knee joint.

## Data Availability

The datasets generated and/or analyzed in the current study are not publicly available due to patient privacy protection but are available from the corresponding author on reasonable request.

## Abbreviations

BS: Bone scintigraphy
SPECT: Single photon emission computed tomography
CT: Computed tomography
PET: Positron emission tomography
SUVs: Standardized uptake values
SD: Standard deviation
WBBS: Whole body bone scintigraphy
^99m^Tc-HDP: Technetium-99m hydroxymethylene diphosphonate
OSEM: Ordered-subset expectation maximization
VOIs: Volumes of interests
ANOVA: One-way analysis of variance
